# Simultaneous Mean and Covariance Correction Filter for Orbit Estimation

**DOI:** 10.3390/s18051444

**Published:** 2018-05-05

**Authors:** Xiaoxu Wang, Quan Pan, Zhengtao Ding, Zhengya Ma

**Affiliations:** 1 School of Automation, Northwestern Polytechnical University, Xi’an 710072, China; quanpan@nwpu.edu.cn (Q.P.); 13623825960@163.com (Z.M.); 2 School of Electrical and Electronic Engineering, The University of Manchester, Manchester M13 9PL, UK; Zhengtao.Ding@manchester.ac.uk

**Keywords:** stochastic dynamic system, nonlinear filter, perturbation identification, simultaneous correction, space target orbit estimation

## Abstract

This paper proposes a novel filtering design, from a viewpoint of identification instead of the conventional nonlinear estimation schemes (NESs), to improve the performance of orbit state estimation for a space target. First, a nonlinear perturbation is viewed or modeled as an unknown input (UI) coupled with the orbit state, to avoid the intractable nonlinear perturbation integral (INPI) required by NESs. Then, a simultaneous mean and covariance correction filter (SMCCF), based on a two-stage expectation maximization (EM) framework, is proposed to simply and analytically fit or identify the first two moments (FTM) of the perturbation (viewed as UI), instead of directly computing such the INPI in NESs. Orbit estimation performance is greatly improved by utilizing the fit UI-FTM to simultaneously correct the state estimation and its covariance. Third, depending on whether enough information is mined, SMCCF should outperform existing NESs or the standard identification algorithms (which view the UI as a constant independent of the state and only utilize the identified UI-mean to correct the state estimation, regardless of its covariance), since it further incorporates the useful covariance information in addition to the mean of the UI. Finally, our simulations demonstrate the superior performance of SMCCF via an orbit estimation example.

## 1. Introduction

The orbit estimation problem is to obtain an accurate estimation of a space target’s (e.g., satellite) position and velocity from noisy observations. The dynamic model of a space target is given below by considering $J_{2}$ perturbations [1]:

$$\ddot{r}=-\frac{\mu }{R^{3}}r+F_{p}+w,$$

where $r={\left[x,y,z\right]}^{T}$ is the position of the space target in the inertial coordinate frame (*I*-*J*-*K*), $R=\sqrt{x^{2}+y^{2}+z^{2}}$, *w* is the white Gaussian noise process, and $F_{p}$ is the instantaneous acceleration due to the $J_{2}$ perturbation [2]. $J_{2}$ is the dimensionless second zonal harmonic that quantifies the major oblateness effect of the Earth. Specially, the $J_{2}$ perturbation may cause a noticeable precession of low-earth orbit (LEO) satellites, and makes the orbital dynamics model to be a strongly nonlinear function. Thus, solving the orbit estimation problem essentially depends on designing a kind of nonlinear state estimation schemes.

**Figure 1:**
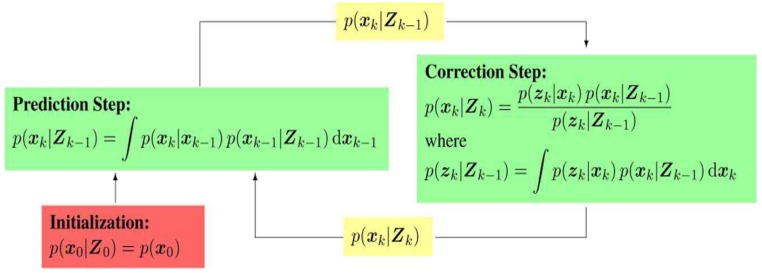
Bayesian inference framework.

At present, nonlinear estimation schemes (NESs) are generally developed from a Bayesian inference framework (BIF) [3] and employed as the widely accepted methods for estimating the space target orbital state. The BIF framework structure is given in Figure 1.

Obviously, to implement BIF in order to estimate the orbit state, NES requires one to compute the strongly nonlinear integral of the perturbation. As an example for Gaussian filters [4]:

$$\int F_{p}p\left(r_{k-1}|Z_{k-1}\right)dr_{k-1},$$

where $p(r_{k-1}|Z_{k-1})$ is assumed to be a Gaussian distribution, and $Z_{k-1}$ denotes the sequence of measurements. The numerical computation of such an integral is both complicated and intractable, such that orbit estimation accuracy may be weak and cannot meet real-time performance requirements. For orbit tracking of a space target, a data-starved and uncertain state evolution [5] requires that the tracking algorithm should be rapid and high-precision.

1. Here, data-starved does not mean a lack of measurements. Rather, it implies that due to the large number of space objectives, the collection, association, processing, and fusion of the measurements needs a lot of time. Hence, the measurement update rate and period are relatively elongated. This requires the tracking algorithm deals with data as efficiently and rapidly as possible.
2. Under a data-starved environment or long data update period, as a result of the consistent long-term (usually several orbital periods) propagation of state uncertainties in high fidelity physics models between measurement updates, a state which is initially Gaussian will inevitably become significantly non-Gaussian or uncertain if propagated over a sufficiently long time span. This requires that the tracking algorithm should be accurate when estimating the uncertain or non-Gaussian state.

The traditional NES, based on BIF, does not meet the rapidity and high-precision requirements due to the complicated and intractable nonlinear perturbation integral.

In order to avoid the nonlinear perturbation integral computation, a joint estimation and identification scheme (JEIS) simply views $F_{p}$ as an additional unknown input (UI) and analytically identifies $F_{p}$ to accurately correct the orbit state estimation [6]. Such analytical identification in JEIS decreases the algorithm complexity while improving the orbit estimation accuracy to some degree by feedback correction. In other words, this paper mainly aims to transfer the intractable nonlinear perturbation integral issue into that of perturbation (viewed as UI) identification, to simplify the orbit estimation algorithm complexity and improve its accuracy.

**Figure 2:**
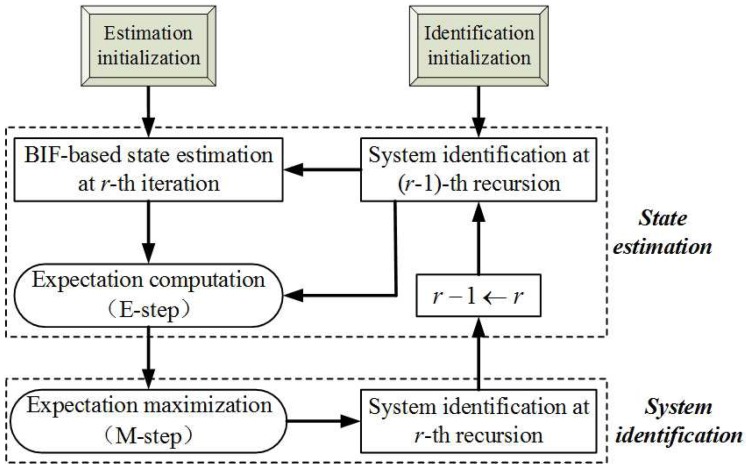
Schematic of the expectation maximization (EM) algorithm, where BIF refers to the Bayesian inference framework.

As a classical representative of JEIS, the expectation maximization (EM) algorithm has received continuous attention and research. The EM framework fits in with the requirements of JESI, as shown in Figure 2. However, the existing EM algorithms always model the UI as a constant variable, independent of the state [6], and only identify the mean of the UI. In the $J_{2}$ perturbation, $F_{p}$ (viewed as UI in JEIS) depends on the state, so it requires at least the first two moments (FTM), i.e., the mean and covariance. To further improve the accuracy of orbit estimation, it is important to research the novel EM algorithm; to identify the UI-FTM (i.e., perturbation-FTM) which simultaneously corrects the state estimation and its covariance. Unfortunately, the existing EM is incapable of dealing with the case when the UI is associated with the state.

**Figure 3:**
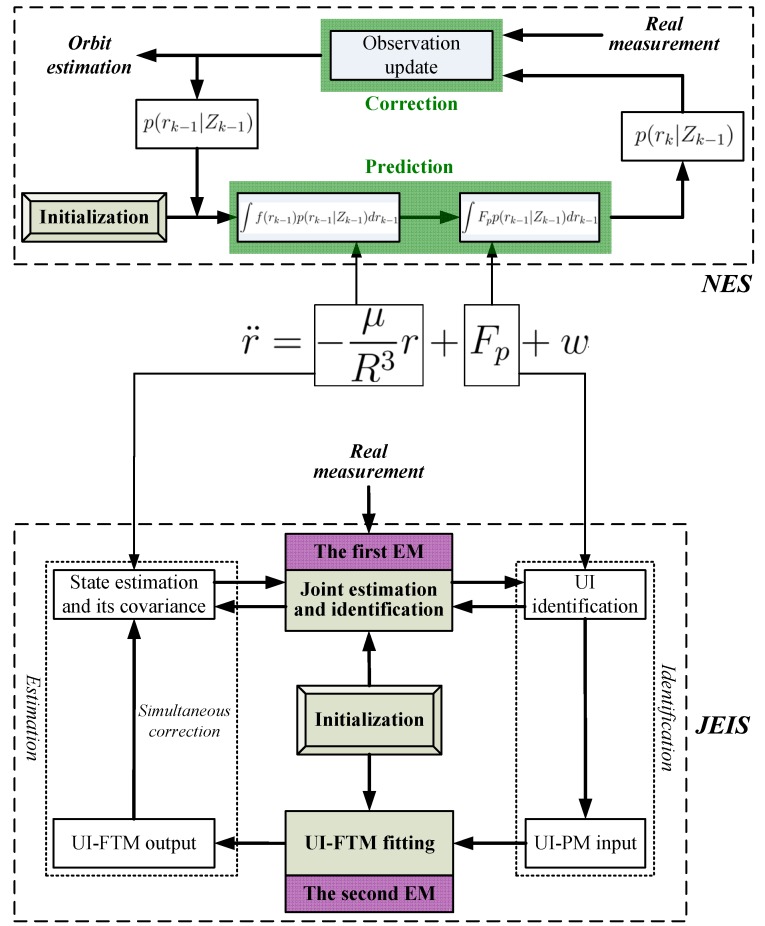
Two-stage EM algorithm.

**Figure 4:**
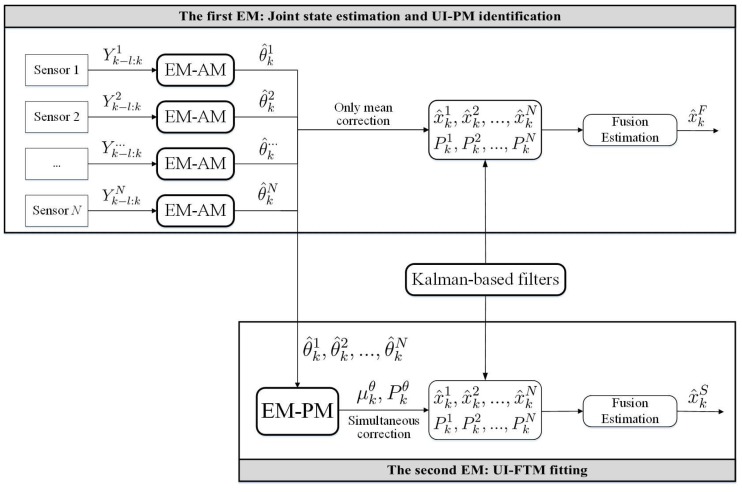
The basic building blocks of the simultaneous mean and covariance correction filter (SMCCF).

Following this idea, that the original orbit estimation is transferred into JESI, we design a novel two-stage EM algorithm (Figure 3 and Figure 4) to deal with the key difficulty of how to simultaneously fit or identify the FTM of UI associated with the state. The main contributions are that: (1) The first EM executes joint orbit state estimation and pseudo measurement (PM) identification using networked multi-sensor observations. PMs can be understood as the indirect reflection of UI from the real observed measurements. (2) The second EM is designed for fitting the UI-FTM by UI-PMs from different sensors, and then used to simultaneously correct the orbit state estimation and its covariance in the first EM. Finally, (3) the identification and fitting computations in these two EMs are analytical, which contributes to rapid and efficient implementation. The simultaneous correction of UI-FTM to the state estimate, as shown in Figure 3, improves the orbit state estimation accuracy. By doing this, a novel simultaneous mean and covariance correction filter (SMCCF) is proposed and achieved. Indeed, vastly different from the standard EM-based filtering scheme, which only estimates the mean characteristic of UI, the novel SMCCF scheme further determines the covariance characteristic of UI. In other words, the joint properties (i.e., both mean and covariance) characterizing the UI in the new scheme provide richer information compared to the single one (i.e., only mean) of the standard approach, which contributes to improved state estimation.

**Figure 5:**
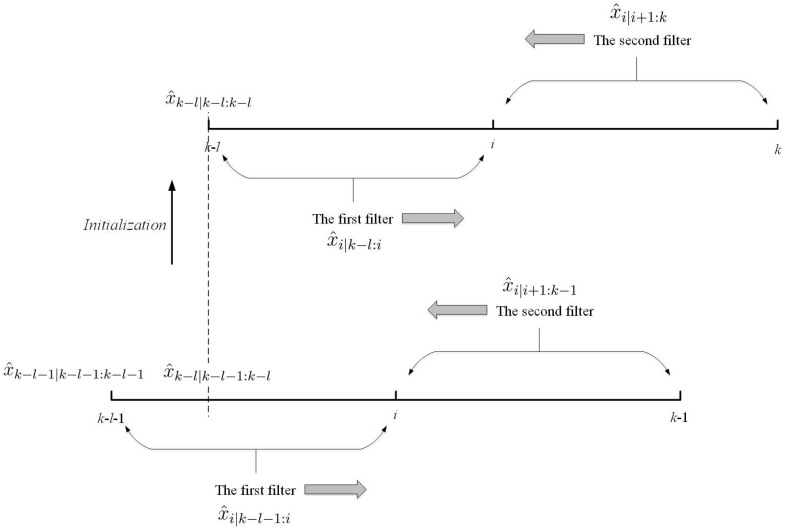
The two-filter smoother and its initialization in EM-AM.

**Figure 6:**
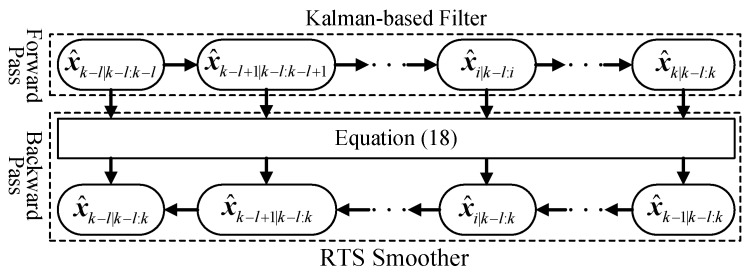
The forward-backward smoother.

**Figure 7:**
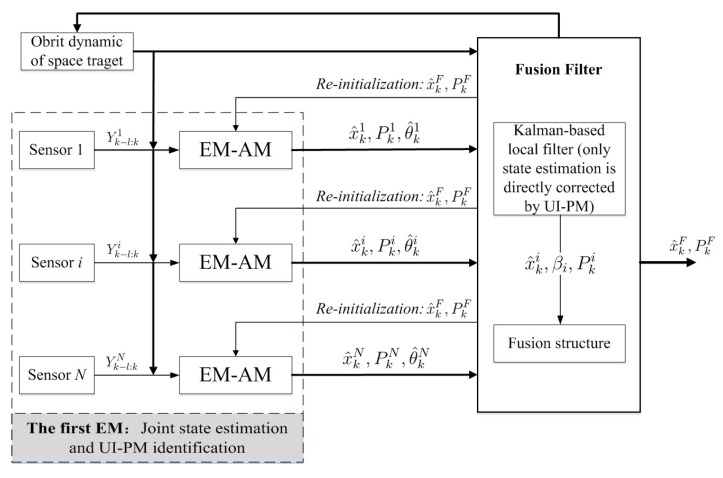
Direct estimation fusion from EM-AM.

**Figure 8:**
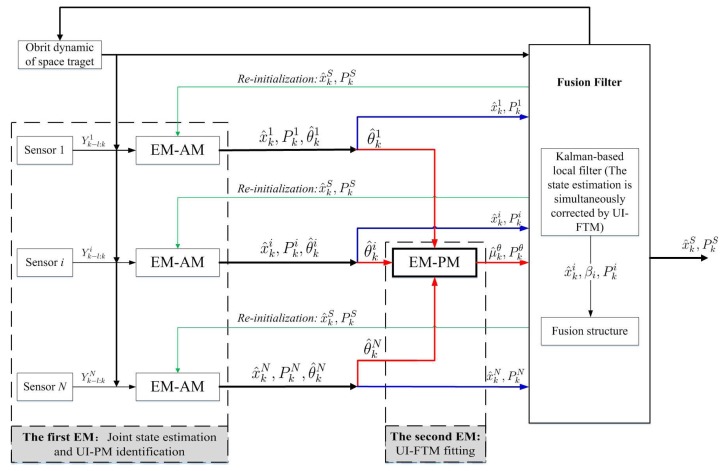
SMCCF.

**Figure 9:**
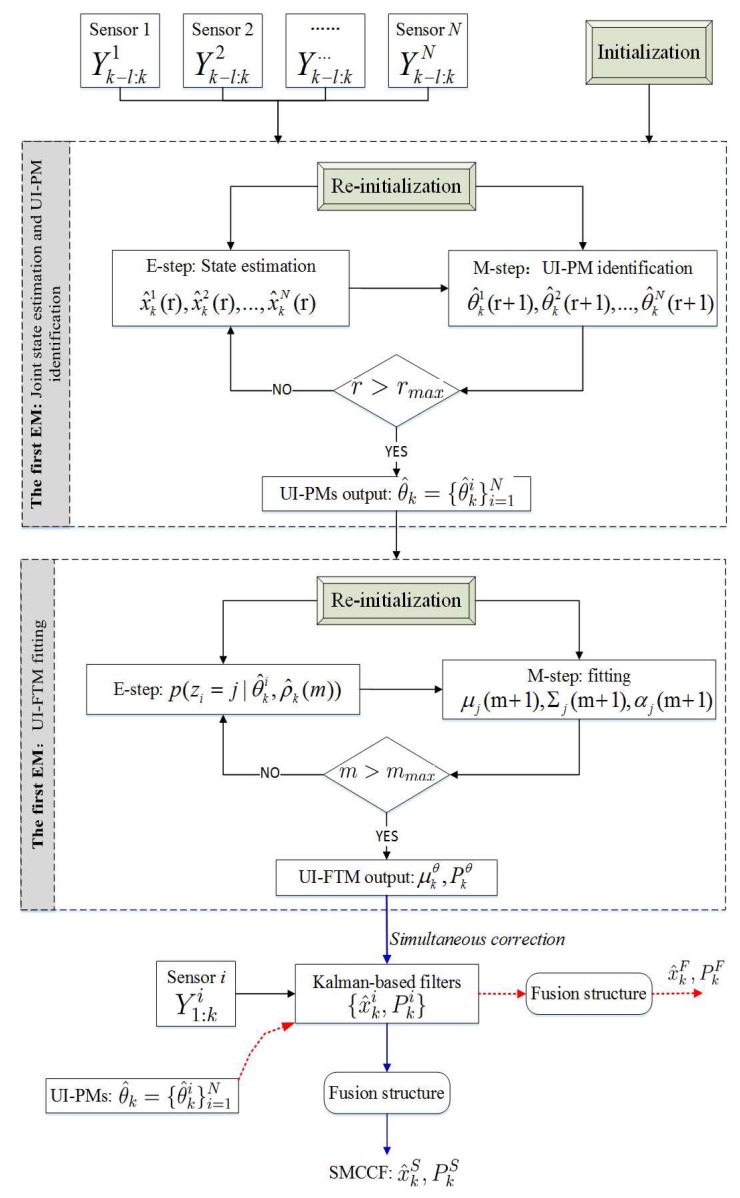
SMCCF execution program.

In this paper, the first EM is called the EM-AM since from the signal input viewpoint it mines the actual measurements (AMs) to characterize the UI, as shown in Figure 3. Similarly, we call the second EM as the EM-PM since it utilizes the PM as input to further fit the UI-FTM. However, different from Reference [7], the expanded contributions of this paper lie in that: (1) we specifically define the pseudo measurement and explain its physical implication (see the test following Equation(19)); (2) initialization of EM-AM plays a key role on the SMCCF performance, so we refine initialization of the EM-AM by using a relatively simple forward–backward smoother, instead of the previous two-filters (see Figure 5 and Figure 6, and Remarks 2 and 3); (3) we elaborate the relationship between EM-AM and EM-PM (see Figure 7 and Figure 8), and the algorithm execution program of SMCCF (see  Figure 9 in Section V-A), in order to facilitate application of SMCCF in practical engineering; and (4) the performance of SMCCF is thoroughly demonstrated by a space target orbit estimation problem and compared to the present classical methods (see Section VI-B). Also different from other EM-based UI identification issues, for example Reference [6], which are only concerned with the mean of UI, this paper designs the novel SMCCF which simultaneously fits the UI-FTM.

This paper is organized as follows. Section 2 formulates the problem. Section 3 and Section 4 design the first and second EMs in the two-stage EM algorithm, respectively. Section 5 gives the fusion structure for combining the two EMs and achieving SMCCF. Section 6 demonstrates the superiority of SMCCF to the standard NES, including the extended Kalman filter (EKF) and the cubature Kalman filter (CKF), by an elaborate simulation of a space target orbit estimation. Finally, some conclusions are drawn in Section 7, including the future work.

*Notations*. The superscripts -1 and T represent the inverse and transpose operations of a matrix, respectively. If *X* is a positive semi-definite or positive definite matrix, we simply write X≥0 or X>0. N(x;μ,Σ) denotes the variable *x* obey a Gaussian distribution with mean μ and covariance Σ. p(⋅) is the probability, for example p(A|B) describes the conditional probability of the variable *A* on *B*. E(⋅) and E(⋅|⋅) represent the expectation or conditional expectation, respectively. We define two operations $D\left(x,P\right)=x^{T}Px$ and $C\left(x\right)=xx^{T}$. The symbols $\hat{\;}$ and $\tilde{\;}$ on top of a random variable, represent an estimate and its error, respectively. For example, $\hat{x}$ denotes the estimate of variable *x* and its estimation error is $\tilde{x}=x-\hat{x}$. Tr(⋅) denotes the trace of matrix.

## 2. Problem Formulation

Consider a general discrete-time stochastic system with additive UIs in the dynamic and measurement models:

(1) $$\begin{array}{c} \left\{\begin{array}{c} x_{k}=f\left(x_{k-1}\right)+w_{k-1}+M_{k-1}a_{k-1}\\ y_{k}=h\left(x_{k}\right)+v_{k}+N_{k}b_{k}\; \end{array}\right. \end{array}$$

where $x\in R^{n},y\in R^{m}$ represent the state and measurement vectors, and f(·),andh(·) are the known dynamic and measurement functions, respectively. The matrices $M\in \;R^{n\times p}$, and $N\in R^{n\times q}$ are known. $w_{k}\;\in \;R^{n}\;\;\;$ and $\;\;\;v_{k}\in R^{m}$ are uncorrelated, zero-mean Gaussian white noise terms satisfying $E\left[w_{k}w_{l}^{T}\right]\;=\;Q_{k}\delta_{kl}$ and $E\left[v_{k}v_{l}^{T}\right]=R_{k}\delta_{kl}$, where $\delta_{kl}$ is the Kronecker delta function. The initial state $x_{0}$ is a Gaussian vector with mean ${\hat{x}}_{0}$ and covariance $P_{0}$. Here, $w_{k},v_{k}$, and $x_{0}$ are mutually independent. The parameters $a_{k}\in R^{p}$, and $b_{k}\in R^{q}$ are unknown UIs which are associated with the state. Also, we set $\theta_{k}=\left\{a_{k},b_{k}\right\}\;$.   

**Remark** **1.** *The model in Equation (1) is common in practical engineering. Consider the example of space object tracking, whose dynamic model is formulated as follows:*

(2) $$\left[\;\begin{array}{c} \ddot{x}\\ \ddot{y}\\ \ddot{z} \end{array}\;\right]\;\;\;=\;\;\;-\;\;\frac{\mu }{r^{3}}\;\left[\;\begin{array}{c} x\\ y\\ z \end{array}\;\right]\;\;\;+\;\;\;\;\frac{\mu }{r^{3}}\;J_{2}\;{\left(\frac{R_{e}}{r}\right)}^{2}\;\left[\;\begin{array}{c} x\left(7.5\frac{z^{2}}{r^{2}}-1.5\right)\\ y\left(7.5\frac{z^{2}}{r^{2}}-1.5\right)\\ z\left(7.5\frac{z^{2}}{r^{2}}-4.5\right) \end{array}\;\right].$$

*The last term in Equation (2) is known as the $J_{2}$ perturbation, $F_{p}$, which is obviously associated with the state. If we regard the perturbation as UI, space object tracking can be modeled by Equation (1). Another example is tracking a maneuvering non-cooperative target, whose dynamic model, due to the modeling error, can be represented by:*

(3) $$x_{k}=\left(F+\Delta F\right)x_{k-1}+w_{k-1}.$$

*If $\Delta Fx_{k-1}$ is considered as the UI, which is obviously coupled with the state, we can also formulate maneuvering target tracking in terms of the model in Equation (1).*

Due to the characteristic that the UI $\theta_{k}$ is highly coupled with the system state, $\theta_{k}$ possesses the FTM. In this case, if we only consider the UI’s mean to correct the state, the estimation accuracy may be undesirable. A feasible method to improve the accuracy is to correct the state by using both the UI-FTM simultaneously. Hence, our objective is to not only identify the mean but also to fit the covariance of the UI. Unfortunately, the existing methods, such as JESI or the standard EM, always regard the UI as a constant. So they all ignore the covariance property of UI, which limits to solving the state estimation problem corresponding to Equation (1).

Combining Equations (1) and (2), we view the perturbation in space target orbit estimation or in tracking as a UI associated with the state. Our aim is to explore the novel EM algorithm for fitting the UI-FTM based on the actual measurements (AMs) $Y_{k-l:k}^{i}\;=\;\left\{y_{k-l}^{i},\cdots ,y_{k}^{i}\right\}\;$(i=1,2,⋯,N) from multi-sensors, where *l* represents the sliding window length. Furthermore, the orbit state estimation is simultaneously corrected by using the fitted UI-FTM.

Different from the classical EM algorithm in Reference [6], a two-stage EM scheme is proposed, as shown in Figure 4 which is a further refinement of Figure 3. In the first stage, for each sensor with $Y_{k-l:k}^{i}$, EM carries out joint state estimation and UI-PM identification, where PM refers to pseudo measurement. *N* sensors need *N* EMs, which run in parallel. All EMs output two groups; the state estimation $\left\{{\hat{x}}_{k}^{1},\;{\hat{x}}_{k}^{2},\cdots ,\;{\hat{x}}_{k}^{N}\right\}$ and the UI-PM identification $\left\{{\hat{\theta }}_{k}^{1},\;{\hat{\theta }}_{k}^{2},\cdots ,{\hat{\theta }}_{k}^{N}\right\}$, where $\left\{{\hat{x}}_{k}^{i},\;{\hat{\theta }}_{k}^{i}\right\}$ corresponds to the *i*-th sensor or the *i*-th EM. Indeed, the EM in the first stage reflects or characterizes UI by the output PM from the input AM. Thus, we also call the first EM as EM-AM, from a signal input viewpoint. The state estimation ${\hat{x}}_{k}^{i}$ in EM-AM running process is only corrected by the PM ${\hat{\theta }}_{k}^{i}$. In the second stage, we further fit the UI-PM by a Gaussian mixture distribution with mean $\mu_{k}^{\theta }$ and covariance $P_{k}^{\theta }$, which can be fed back into the first EM to jointly correct the state estimation and its covariance. Similarly, we name the second EM as EM-PM. By linking these two EMs, the SMCCF is proposed by designing the fusion structure. From a sense of whether enough information is mined or utilized, the ${\hat{x}}_{k}^{S}$ by SMCCF should be superior to the ${\hat{x}}_{k}^{F}$ by the existing nonlinear filters or algorithms in accuracy, since the former further mines covariance information in addition to the mean of UI.

Following Figure 4, Section 3 and Section 4 derive the EM-AM and EM-PM, respectively. Section 5 further links these two kinds of EMs, and clarifies the single input-output relationship between them, by designing a new fusion structure. By doing this, the SMCCF is achieved.

## 3. EM-AM

The EM algorithm was introduced by Dempster et al. [8] to solve maximum likelihood (ML) estimation with incomplete or missing data. If we consider the state in Equation (1) as the missing data and $\theta_{k}$ as the parameters which need to be identified, then the EM framework [9] is suitable for joint state estimation and UI-PM identification precisely.

To implement the EM-AM algorithm in each sensor in Figure 4, we first need to compute the conditional expectation, $Q(\theta ,{\hat{\theta }}_{r})$, of the complete data log-likelihood function $L_{\theta }\left(X_{k-l}^{k},Y_{k-l}^{k}\right)$ in the E-step. Before addressing this, by first using Bayes’ rule and the Markov property of the model in Equation (1), we have:

(4) $$L_{\theta }\left(X_{k-l-1}^{k},\;Y_{k-l}^{k}\right)=\log p_{\theta }\left(X_{k-l-1}^{k},\;Y_{k-l}^{k}\right)=\log p_{\theta }\left(x_{k-l-1}\right)+\sum_{i=k-l}^{k}\log p_{\theta }\left(x_{i}|x_{i-1}\right)+\sum_{i=k-l}^{k}\log p_{\theta }\left(y_{i}|x_{i}\right)\;.$$

Then, applying the following Gaussian approximation:

(5) $$\begin{array}{c} \begin{array}{c} p_{\theta }\left(x_{i}|x_{i-1}\right)=N\left(x_{i}-f\left(x_{i-1}\right)-M_{i-1}a_{i-1};0,Q_{i-1}\right) \end{array} \end{array}$$

(6) $$\begin{array}{c} \begin{array}{c} p_{\theta }\left(y_{i}|x_{i}\right)=N\left(y_{i}-h\left(x_{i}\right)-N_{i}b_{i};0,R_{i}\right), \end{array} \end{array}$$

Equation (4) can be rearranged to yield:

(7) $$\begin{array}{c} L_{\theta }\left(X_{k-l-1}^{k},Y_{k-l}^{k}\right)=L_{0}^{}+L_{1}^{}+L_{2}^{}+L_{3}^{} \end{array}$$

where $L_{0}$ is a constant independent of $\theta_{k}$, and is thus omitted, and:

(8) $$L_{1}=\log p_{\theta }\left(x_{k-l-1}\right)$$

(9) $$L_{2}^{}=-\frac{1}{2}\sum_{i=k-l}^{k}D(x_{i}\;-\;f\left(x_{i\;-\;1}\right)\;-\;M_{i\;-\;1}a_{i-1},Q_{i\;-\;1})$$

(10) $$L_{3}^{}=-\frac{1}{2}\sum_{i=k-l}^{k}D(y_{i}-h\left(x_{i}\right)-N_{i}b_{i},R_{i}).$$

The following subsections outline the steps to achieve EM-AM according to Figure 2; the E-step and M-step.

### 3.1. E-Step in EM-AM

Taking the expectation of Equation (7) with respect to the probability density function $p_{{\hat{\theta }}_{r}}\left(X_{k-l-1}^{k}|Y_{k-l}^{k}\right)$, we can obtain:

(11) $$Q\left(\theta ,{\hat{\theta }}_{r}\right)=E_{{\hat{\theta }}_{r}}\left[L_{\theta }\left(X_{k-l}^{k},\;Y_{k-l}^{k}\right)|Y_{k-l}^{k}\right]=\int L_{\theta }\left(X_{k-l-1}^{k},\;Y_{k-l}^{k}\right)\cdot p_{{\hat{\theta }}_{r}}\left(X_{k-l-1}^{k}|Y_{k-l}^{k}\right)dX_{k-l-1}^{k}=I_{0}+I_{1}+I_{2}+I_{3},$$

where $I_{0}$ is a constant corresponding to $L_{0}$. Note that this posterior probability, $p_{{\hat{\theta }}_{r}}\left(X_{k-l-1}^{k}|Y_{k-l}^{k}\right)$, corresponds to the state estimation on knowing the identification value ${\hat{\theta }}_{r}$ at the *r*-th iteration of EM-AM. It can be computed by Kalman-based estimators, such as Gaussian filters including EKF, and CKF, among others. Thus, we have:

(12) $$I_{1}=E_{{\hat{\theta }}_{r}}\left[L_{1}^{}|Y_{k-l}^{k}\right]=\;\int \;\log p_{\theta }\left(x_{k\;-\;l\;-\;1}\right)\;\cdot \;p_{{\hat{\theta }}_{r}}\left(x_{k\;-\;l\;-\;1}|Y_{k\;-\;l}^{k}\right)dx_{k\;-\;l\;-\;1}$$

(13) $$I_{2}=E_{{\hat{\theta }}_{r}}\left[L_{2}^{}|Y_{k-l}^{k}\right]=-\frac{1}{2}Tr\left\{\xi_{I_{2}}^{k}-\sum_{i=k-l}^{k}Q_{i-1}^{-1}P_{i,i|k-l:k}\right\}$$

$$\xi_{I_{2}}^{k}\;\;=\;\;\;\sum_{i\;=\;k\;-\;l}^{k}\;\;Q_{i\;-\;1}^{\;-\;1}\;\int C\left[{\hat{x}}_{i|k-l:k}-f\left(x_{i-1}\right)-M_{i-1}a_{i-1})\right]p_{{\hat{\theta }}_{r}}\left(X_{k-l}^{k}|Y_{k-l}^{k}\right)dx_{i-1}$$

(14) $$I_{3}=E_{{\hat{\theta }}_{r}}\left[L_{3}^{}|Y_{k-l}^{k}\right]=-\frac{1}{2}Tr\left\{\xi_{I_{3}}^{k}\right\}$$

$$\xi_{I_{3}}^{k}=\sum_{i\;=\;k\;-\;l}^{k}R_{i}^{\;-\;1}\;\int C\left[y_{i}-h\left(x_{i}\right)-N_{i}b_{i}\right]p_{{\hat{\theta }}_{r}}\left(X_{k-l}^{k}|Y_{k-l}^{k}\right)dx_{i},\;$$

where the computations of Equations (13) and (14) refer to:

$$\begin{array}{cc} E_{{\hat{\theta }}_{r}}\left[{\tilde{x}}_{i,i|k-l:k}{\left(f\left(x_{i-1}\right)+M_{i-1}a_{i-1}\right)}^{T}|Y_{k-l}^{k}\right] & =E_{{\hat{\theta }}_{r}}\left[\left(f\left(x_{i-1}\right)+M_{i-1}a_{i-1}\right){\tilde{x}}_{i,i|k-l:k}^{T}|Y_{k-l}^{k}\right]\\ & =E_{{\hat{\theta }}_{r}}\left[{\tilde{x}}_{i,i|k-l:k}{\left(x_{i}-w_{i-1}\right)}^{T}|Y_{k-l}^{k}\right]\\ & =P_{i,i|k-l:k} \end{array}$$

$$\begin{array}{cc} \xi_{Bk} & =\sum_{i=k-l}^{k}Q_{i-1}^{-1}E_{{\hat{\theta }}_{r}}\left[C\left(x_{i}-f\left(x_{i-1}\right)-M_{i-1}a_{i-1}\right)|Y_{k-l}^{k}\right]\\ & =\sum_{i=k-l}^{k}Q_{i-1}^{-1}E_{{\hat{\theta }}_{r}}\left[C\left(\left({\hat{x}}_{i}+{\tilde{x}}_{i}\right)-f\left(x_{i-1}\right)-M_{i-1}a_{i-1}\right)|Y_{k-l}^{k}\right]\\ & =\sum_{i=k-l}^{k}Q_{i-1}^{-1}E_{{\hat{\theta }}_{r}}\left[C\left({\hat{x}}_{i}-f\left(x_{i-1}\right)-M_{i-1}a_{i-1}\right)|Y_{k-l}^{k}\right]-\sum_{i=k-l}^{k}Q_{i-1}^{-1}P_{i,i|k-l:k} \end{array}$$

$$\begin{array}{cc} \xi_{Ck} & =\sum_{i=k-l}^{k}R_{i}^{-1}E_{{\hat{\theta }}_{r}}\left[C\left(y_{i}-h\left(x_{i}\right)-N_{i}b_{i}\right)|Y_{k-l}^{k}\right]\\ & =\sum_{i=k-l}^{k}R_{i}^{-1}\int C\left(y_{i}-h\left(x_{i}\right)-N_{i}b_{i}\right)p_{{\hat{\theta }}_{r}}\left(x_{i}|Y_{k-l}^{k}\right)dx_{i}, \end{array}$$

and ${\hat{x}}_{i|k-l:k}$ and $P_{i,i|k-l:k}$ are the posterior estimates of the state $x_{i}$ under the measurements $Y_{k-l}^{k}$ from the interval [k−l, *k*] with the minimum mean square error (MMSE) criterion:

(15) $$\begin{array}{c} {\hat{x}}_{i|k-l:k}=E_{{\hat{\theta }}_{r}}\left[x_{i}|Y_{k-l}^{k}\right]=\int x_{i}p_{{\hat{\theta }}_{r}}\left(x_{i}|Y_{k-l}^{k}\right)dx_{i} \end{array}$$

(16) $$\begin{array}{cc} & P_{i,i|k-l:k}=E_{{\hat{\theta }}_{r}}\left[{\tilde{x}}_{i|k-l:k}{\tilde{x}}_{i|k-l:k}^{T}|Y_{k-l}^{k}\right]=\int x_{i}x_{i}^{T}p_{{\hat{\theta }}_{r}}\left(x_{i}|Y_{k-l}^{k}\right)dx_{i}-{\hat{x}}_{i|k-l:k}{\hat{x}}_{i|k-l:k}^{T}. \end{array}$$

In Equations (8) and (12), the terms $L_{1}$ and $I_{1}$ are associated with the UI θ, but θ is hidden in the probability $p_{\theta }\left(x_{k-l-1}\right)$. Hence, we can not obtain the dominant expressions of $L_{1}$ and $I_{1}$ like we can for $L_{2}$, $L_{3}$, $I_{2}$, and $I_{3}$. In order to compute the dominant derivative of $Q(\theta ,{\hat{\theta }}_{r})$ with respect to θ, we have to ignore $L_{1}$ and $I_{1}$. But ignoring $L_{1}$ and $I_{1}$ is reasonable in some sense, because: (1) if l=k−1, then $L_{1}$ equals $\log p_{\theta }\left(x_{0}\right)$, which is independent of θ, and (2) since the UI is associated with the state in our problem, θ is time-varying. In order to quickly reflect or sketch this time-varying UI, it should gradually forget the previous information while preserving the current information in a sliding window of length *l*. This means that the previous measurements in interval [1,k−l−1] are given up.

In order to compute $Q(\theta ,{\hat{\theta }}_{r})$, the state estimation ${\hat{x}}_{i|k-l:k}$ and its covariance $P_{i,i|k-l:k}$ in the interval [k−l,k] must be computed first, which is known as a smooth problem and can be implemented by using a fixed interval smoother. One example is the two-filter smoother [10], which is formulated as follows:

(17) $$\begin{array}{c} \left\{\;\;\;\;\begin{array}{c} {\hat{x}}_{i|k\;-\;l:k}\;\;\;=\;\;\;P_{i\;,i|k\;-\;l:k}\;\left[{\left(P_{i\;,i|k\;-\;l:i}\right)}^{\;-\;1\;}{\hat{x}}_{i|k\;-\;l:i}\;\;+\;\;{\left(P_{i\;,i|i\;+\;1:k}\right)}^{\;-\;1\;}{\hat{x}}_{i|i\;+\;1\;:k}\right]\\ P_{i,i|k\;-\;l:k}\;\;=\;\;{\left[{\left(P_{i,i|k\;-\;l:i}\right)}^{-1}\;\;+\;\;{\left(P_{i,i|i\;+\;1:k}\right)}^{-1}\right]}^{-1}\; \end{array}\right. \end{array}$$

where, as shown in Figure 5 the first filter (i.e., ${\hat{x}}_{i|k-l:i}$, $P_{i,i|k-l:i}$) is computed by Kalman-based filtering under the model in Equation (1) and the second filter (i.e., ${\hat{x}}_{i|i+1:k}$, $P_{i,i|i+1:k}$) is also computed with a similar Kalman-based filter but under the inverse form of Equation (1), which runs backwards in time. The unscented Kalman filter (UKS) presented in Reference [11] can be understood as an approximate implementation of this form of smoother in nonlinear systems.

Another type of smoother is the forward-backward smoother [12], names as Rauch-Tung-Striebel (RTS) Smoother:

(18) $$\left\{\begin{array}{c} {\hat{x}}_{i|k-l:k}={\hat{x}}_{i|k-l:i}+A_{i}[{\hat{x}}_{i+1|k-l:k}-{\hat{x}}_{i+1|k-l:i}]\\ P_{i,i|k-l:k}=P_{i,i|k-l:i}+A_{i}[P_{i+1,i+1|k-l:k}-P_{i+1,i+1|k-l:i}]A_{i}^{T}\\ A_{i}=P_{i,i+1|k-l:i}P_{i+1,i+1|k-l:i}^{-1}. \end{array}\right.$$

Here, the forward pass applies Kalman-based filters to compute the filtering estimates ${\hat{x}}_{i|k-l:i}$, $P_{i,i|k-l:i}$ and the predictive estimates ${\hat{x}}_{i+1|k-l:i}$, $P_{i+1,i+1|k-l:i}$, which are same as those in two-filter smoother. But in the backward pass, the smoothing recursion starts from last time step *k* and proceeds backwards in time. That is, to compute the smoothing estimation from ${\hat{x}}_{i+1|k-l:k}$, $P_{i+1,i+1|k-l:k}$ to ${\hat{x}}_{i|k-l:k}$, $P_{i,i|k-l:k}$, which is different from the second filter in the two-filter smoother. A derivation of the structure in Equation (18), can be found in References [13,14,15], and its computation program is shown in Figure 6.

**Remark** **2.** *A key point of implementing Equation (17) is initialization. If we regard the interval [k−l,k] as [1,l+1] (where the initial value is ${\hat{x}}_{0}$ ), the first filter in Equation (17) should be initialized by ${\hat{x}}_{k-l-1|k-l-1:k-l-1}$, which is obtained in the proposed SMCCF by knowing the identified UI-FTM in the interval [k−l−1,k−1]. In more detail, the first filter is easily initialized by a standard Kalman-based filter with the initial state value ${\hat{x}}_{k-l-1|k-l-1:k-l-1}$. The initialization of the second filter depends on that of the first filter. In other words, the second filter also operates a standard Kalman-based filter but under the inverse form of Equation (1) with the initial value ${\hat{x}}_{k|k-l:k}$, which is obtained by the first filter. The details of the second filter are elaborated in Reference [11]. The inverse computation of Equation (1) complicates the two-filter smoother. Specifically for nonlinear systems, it is intractable and even infeasible to obtain the model’s inverse form.*

**Remark** **3.** *In the forward-backward smoother, only the forward filter needs to be initialized, which is similar to the initialization of the first filter in the two-filter smoother. While the backward smoothing computation in Equation (18) proceeds backwards in time, i.e., from ${\hat{x}}_{k|k-l:k}$ to ${\hat{x}}_{k-l|k-l:k}$. Note that ${\hat{x}}_{k|k-l:k}$ is obtained by the forward filter. Hence, from the viewpoint of a simpler initialization, the forward-backward smoother outperforms the two-filter one in operation. More importantly, the former contributes to simply achieving the following M-step in EM-AM, without computing the inverse form of the nonlinear model. Due to the above two reasons, this paper implements the forward-backward smoother for the state estimation in EM-AM.*

### 3.2. M-Step in EM-AM

So far, we have completed the computation of the expectation $Q(\theta ,{\hat{\theta }}_{r})$ with respect to θ by the E-step. Before maximizing $Q(\theta ,{\hat{\theta }}_{r})$, the pseudo measurement (PM) of UI is defined as follows:

(19) $${\hat{\theta }}_{PM}=\int \theta p_{{\hat{\theta }}_{r}}\left(X_{k-l}^{k}|Y_{k-l}^{k}\right)dX_{k-l}^{k}\;,$$

where θ is coupled to the state, but the inherent coupling characteristic is unknown. Equation (19) indeed implies the posterior identification of UI under actual measurements, i.e., that $Y_{k-l}^{k}$ affects the state estimation $p_{{\hat{\theta }}_{r}}\left(X_{k-l}^{k}|Y_{k-l}^{k}\right)$ and further acts on the UI identification (${\hat{\theta }}_{PM}$). Hence PM’s definition concretely links estimation and identification in EM-AM.

The M-step requires a maximization of $Q(\theta ,{\hat{\theta }}_{r})$ with respect to θ. Here, we let the first derivatives of $Q(\theta ,{\hat{\theta }}_{r})$ with respect to θ to be zero directly:

(20) $$\begin{array}{c} \begin{array}{c} \frac{\partial Q(\theta ,{\hat{\theta }}_{r})}{\partial a}=\sum_{i=k-l}^{k}M_{i-1}^{T}Q_{i-1}^{-1}\left[{\hat{x}}_{i|k-l:k}-\int f\left(x_{i-1}\right)\cdot p_{{\hat{\theta }}_{r}}\left(x_{i-1}|Y_{k-l}^{k}\right)dx_{i-1}-M_{i-1}\int ap_{{\hat{\theta }}_{r}}\left(X_{k-l}^{k}|Y_{k-l}^{k}\right)dX_{k-l}^{k}\right]=0 \end{array} \end{array}$$

(21) $$\begin{array}{c} \begin{array}{c} \frac{\partial Q(\theta ,\theta_{k})}{\partial b}=\sum_{i=k-l}^{k}N_{i}^{T}R_{i}^{-1}\left[y_{i}-\int h\left(x_{i}\right)p_{{\hat{\theta }}_{r}}\left(x_{i}|Y_{k-l}^{k}\right)dx_{i}-N_{i}\int bp_{{\hat{\theta }}_{r}}\left(X_{k-l}^{k}|Y_{k-l}^{k}\right)dX_{k-l}^{k}\right]=0. \end{array} \end{array}$$

Thus, we have:

(22) $$\begin{array}{c} {\hat{a}}_{PM}=A_{k}\times \left[\sum_{i=k-l}^{k}M_{i-1}^{T}Q_{i-1}^{-1}\left({\hat{x}}_{i|k-l:k}-\int f\left(x_{i-1}\right)\cdot p_{{\hat{\theta }}_{r}}\left(x_{i-1}|Y_{k-l}^{k}\right)dx_{i-1}\right)\right] \end{array}$$

(23) $${\hat{b}}_{PM}=B_{k}\left[\sum_{i=k-l}^{k}N_{i}^{T}R_{i}^{-1}\left(y_{i}-\int h\left(x_{i}\right)p_{{\hat{\theta }}_{r}}\left(x_{i}|Y_{k-l}^{k}\right)dx_{i}\right),\right]$$

where:

$$A_{k}={\left(\sum_{i=k-l}^{k}M_{i-1}^{T}Q_{i-1}^{-1}M_{i-1}\right)}^{-1}$$

$$B_{k}={\left(\sum_{i=k-l}^{k}N_{i}^{T}R_{i}^{-1}N_{i}\right)}^{-1}.$$

In other words, from Equation (20) to (23), PM can be understood as an average and indirect measurement of UIs by allowing actual observations to further influence UI-PM identification. The derivatives in Equations (20) and (21) essentially treat UIs (*a* and *b*) as kinds of average values in the interval [k−l,k]. Thus UI-PM identification in Equations (22) and (23) indeed determines the estimated values ${\hat{a}}_{r+1}$ and ${\hat{b}}_{r+1}$ at the r+1-th iteration:

$${\hat{a}}_{r+1}={\hat{a}}_{PM},\;{\hat{b}}_{r+1}={\hat{b}}_{PM}.$$

Further, the second derivatives of $Q(\theta ,{\hat{\theta }}_{r})$ with respect to θ:

(24) $$\;\frac{\partial^{2}Q\left(\theta ,{\hat{\theta }}_{r}\right)}{\partial a^{2}}=-\sum_{i=k-l}^{k}M_{i-1}^{T}Q_{i-1}^{-1}M_{i-1}<0$$

(25) $$\;\frac{\partial^{2}Q\left(\theta ,{\hat{\theta }}_{r}\right)}{\partial b^{2}}=-\sum_{i=k-l}^{k}N_{i}^{T}R_{i}^{-1}N_{i}<0,$$

are strictly negative-definite only if $Q_{i}>0$ and $R_{i}>0$, which can be always guaranteed in practice. This implies that Equations (22) and (23) uniquely maximize the convex $Q(\theta ,{\hat{\theta }}_{r})$. However, Equations (22) and (23) are only formally analytical and optimal since they need to approximately compute the following general integrals:

(26) I=∫g(x)ω(x)dx,

where g(x) generalizes the functions $f(x_{i-1})$ and $h(x_{i})$, while ω(x) describes $p_{{\hat{\theta }}_{r}}\left(x_{i-1}|Y_{k-l}^{k}\right)$ or $p_{{\hat{\theta }}_{r}}\left(x_{i}|Y_{k-l}^{k}\right)$. As mentioned in Remark 3, if we use Kalman-based filters to compute such integrals, for example the Gaussian filter, ω(x) denotes the corresponding Gaussian distribution. So numerical approximations such as EKF, and CKF can implement this integral computation (see References [16,17]). To avoid repetition, the process is omitted.

After identifying the UI-PM, how to further fit the UI-FTM with the PM will be dealt with in designing the EM-PM. According to Figure 4, every sensor runs one EM-AM to output a group of $\left\{{\hat{x}}_{k}^{i},{\hat{\theta }}_{k}^{i}\right\}$. In fact, only ${\hat{x}}_{k}^{i}$ is directly corrected by UI-PM ${\hat{\theta }}_{k}^{i}$ in the EM-AM. The following section designs the EM-PM for fitting the covariance of UI-PM to correct the estimation covariance $P_{k}^{i}$.

**Remark** **4.** *At each recursion, the PM parameter is initialized with the previous PM identification, i.e., ${\hat{\theta }}_{k}$ with ${\hat{\theta }}_{k-1}$. At the beginning time, ${\hat{\theta }}_{0}$ can be initialized by prior information. For example, ${\hat{\theta }}_{0}$ is computed from ${\hat{x}}_{0}$ and $P_{0}$ by performing one recursion of the conventional nonlinear filtering, i.e., through the perturbation nonlinear integral to compute ${\hat{\theta }}_{0}$:*

(27) $${\hat{\theta }}_{0}=\int F_{p}p\left(x_{0}\right)dx_{0},$$

*whose implementation is similar to that in Equation (26).*

## 4. EM-PM

This section will fit the UI-FTM from the identified UI-PM in the probability domain, not the time-domain, by the EM algorithm. The UI-FTM fitting issue is to find the available probability density $p({\hat{\theta }}_{k})$ for describing the stochastic property of UI-PM, ${\hat{\theta }}_{k}\;\;=\;\;{\left\{{\hat{\theta }}_{k}^{i}\right\}}_{i=1}^{N}$, where $p({\hat{\theta }}_{k}^{i})$ corresponds to the *i*-th sensor. It has been proven that a Gaussian mixture (GM) form can approximate any probability density function as closely as desired [16]. In order to determine the underlying probability distribution $p({\hat{\theta }}_{k})$ of the identified UI-PM set, ${\hat{\theta }}_{k}\;\;=\;\;{\left\{{\hat{\theta }}_{k}^{i}\right\}}_{i=1}^{N}$ at time *k*, we assume that the stochastic property of UI-PM is generated using *M* probability distributions where each is a Gaussian function with weight $\alpha_{j}$, mean $\mu_{j}$, and covariance $\Sigma_{j}$:

(28) $$p\left({\hat{\theta }}_{k}|\rho \right)=\sum_{j=1}^{M}\alpha_{j}p_{j}\left({\hat{\theta }}_{k}|\mu_{j},\Sigma_{j}\right)$$

where $\rho ={\left\{\alpha_{j},\mu_{j},\Sigma_{j}\right\}}_{j=1}^{M}$ are the associated parameters with UI-FTM fitting, $\sum_{j=1}^{M}\alpha_{j}=1$, *M* is the number of Gaussian components in the GM, and $p_{j}\left({\hat{\theta }}_{k}|\mu_{j},\Sigma_{j}\right)=N\left({\hat{\theta }}_{k};\mu_{j},\Sigma_{j}\right)$ is a multi-dimensional Gaussian probability density. The UI-FTM fitting is further transferred into the parameter identification of ρ.

A detailed discussion for GM fitting based on the EM framework has been proposed in Reference [19]. Here, we only give a brief derivation about how to apply EM for fitting UI-FTM. First, the log-likelihood function of $p({\hat{\theta }}_{k})$ can be written as:

(29) $$\begin{array}{cc} & L_{\rho }\left({\hat{\theta }}_{k}\right)=\ln p\left({\hat{\theta }}_{k}|\rho \right)=\ln\prod_{i=1}^{N}p({\hat{\theta }}_{k}^{i}|\rho )=\sum_{i=1}^{N}\ln p({\hat{\theta }}_{k}^{i}|\rho )=\sum_{i=1}^{N}\ln\sum_{j=1}^{M}\alpha_{j}p_{j}\left({\hat{\theta }}_{k}^{i}|\vartheta_{j}\right), \end{array}$$

where $\vartheta_{j}=\left\{\mu_{j},\Sigma_{j}\right\}$. Direct maximization of Equation (29) can not be achieved since we don’t know which Gaussian component in the GM is the most suitable for expressing or fitting the probability $p({\hat{\theta }}_{k}^{i})$ of the *j*-th UI-PM . For dealing with this key point, a missing variable $z={\left\{z_{i}\right\}}_{i=1}^{N}$ is introduced, which establishes a simple relation between the UI-PM and Gaussian components. Elaborately, $z_{i}=\left\{1,2,\cdots ,M\right\}≜\Gamma$ and $z_{i}=j$ denotes that the *i*-th PM, ${\hat{\theta }}_{k}^{i}$, is probably characterized by the *j*-th Gaussian component, i.e., $p_{j}\left({\hat{\theta }}_{k}^{i}|\mu_{j},\Sigma_{j}\right)$. By doing this, $\left\{{\hat{\theta }}_{k},z\right\}$ constructs the complete-data set for further establishing the complete-data likelihood function:

(30) $$\begin{array}{c} \ln p\left({\hat{\theta }}_{k},z|\rho \right)=\ln\prod_{i=1}^{N}p\left({\hat{\theta }}_{k}^{i},z_{i}|\rho \right)=\sum_{i=1}^{N}\ln p\left({\hat{\theta }}_{k}^{i},z_{i}|\rho \right)=\sum_{i=1}^{N}\ln\alpha_{z_{i}}p_{z_{i}}\left({\hat{\theta }}_{k}^{i}|\mu_{z_{i}},\Sigma_{z_{i}}\right), \end{array}$$

where the subscript $z_{i}$ denotes all of the Gaussian components which the *i*-th PM, ${\hat{\theta }}_{k}^{i}$, may correspond to. Indeed:

$$\alpha_{z_{i}}=\alpha_{z_{i}=j}$$

$$p_{z_{i}}\left({\hat{\theta }}_{k}^{i}|\mu_{z_{i}},\Sigma_{z_{i}}\right)=p_{z_{i}=j}\left({\hat{\theta }}_{k}^{i}|\mu_{z_{i}=j},\Sigma_{z_{i}=j}\right)$$

with j∈{1,2,⋯,M}. It should be emphasized that ${\hat{\theta }}_{k}^{i}$ and $z_{i}$ have a one-to-one correspondence, which well explains Equation (30).

Then, the E-step needs to evaluate the conditional expectation of $\ln p\left({\hat{\theta }}_{k},z|\rho \right)$ under $p\left(z|{\hat{\theta }}_{k}^{i},{\hat{\rho }}_{m}\right)$:

(31) $$Q\left(\rho |{\hat{\rho }}_{m}\right)=E_{z|{\hat{\theta }}_{k},{\hat{\rho }}_{m}}\ln p\left({\hat{\theta }}_{k},z|\rho \right),$$

where ${\hat{\rho }}_{m}$ is the fitting result of ρ in the *m*-th iteration.

Application of the following equation:

$$p\left(z|{\hat{\theta }}_{k},{\hat{\rho }}_{m}\right)=\prod_{i=1}^{N}p\left(z_{i}|{\hat{\theta }}_{k}^{i},{\hat{\rho }}_{m}\right)$$

to further arrange Equation (31) obtains:

(32) $$\begin{array}{cc} Q(\rho |{\hat{\rho }}_{m}) & =E_{z|{\hat{\theta }}_{k},{\hat{\rho }}_{m}}\ln p\left({\hat{\theta }}_{k},z|\rho \right)\\ & =\sum_{z\in \Gamma }\left[\ln p\left({\hat{\theta }}_{k},z|\rho \right)\right]p\left(z|{\hat{\theta }}_{k},{\hat{\rho }}_{m}\right)\\ & =\sum_{j=1}^{M}\left[\sum_{i=1}^{N}\ln\alpha_{j}p_{j}\left({\hat{\theta }}_{k}^{i}|\mu_{j},\Sigma_{j}\right)\right]p\left(j|{\hat{\theta }}_{k}^{i},{\hat{\rho }}_{m}\right)\\ & =\sum_{j=1}^{M}\sum_{i=1}^{N}\ln\left(\alpha_{j}\right)p\left(j|{\hat{\theta }}_{k}^{i},{\hat{\rho }}_{m}\right)+\sum_{j=1}^{M}\sum_{i=1}^{N}\left[\ln p_{j}\left({\hat{\theta }}_{k}^{i}|\mu_{j},\Sigma_{j}\right)\right]p\left(j|{\hat{\theta }}_{k}^{i},{\hat{\rho }}_{m}\right), \end{array}$$

with:

(33) $$\begin{array}{c} p_{j}\left({\hat{\theta }}_{k}^{i}|\vartheta_{j}\right)=\frac{1}{{\left(2\pi \right)}^{d/2}|\Sigma_{j}{|}^{1/2}}\exp\left\{-\frac{1}{2}{\left({\hat{\theta }}_{k}^{i}-\mu_{j}\right)}^{T}\Sigma_{j}^{-1}\left({\hat{\theta }}_{k}^{i}-\mu_{j}\right)\right\} \end{array}$$

(34) $$\begin{array}{c} \begin{array}{c} p\left(j|{\hat{\theta }}_{k}^{i},{\hat{\rho }}_{m}\right)=p\left(z_{i}=j|{\hat{\theta }}_{k}^{i},{\hat{\rho }}_{m}\right)=\frac{p\left({\hat{\theta }}_{k}^{i},z_{i}=j|{\hat{\rho }}_{m}\right)}{p\left({\hat{\theta }}_{k}^{i}|{\hat{\rho }}_{m}\right)}=\frac{{\hat{\alpha }}_{j,m}p_{j}\left({\hat{\theta }}_{k}^{i}|{\hat{\vartheta }}_{j,m}\right)}{\sum_{s=1}^{M}{\hat{\alpha }}_{s,m}p_{s}\left({\hat{\theta }}_{k}^{i}|{\hat{\vartheta }}_{s,m}\right)} \end{array} \end{array}$$

(35) $$\begin{array}{c} p_{j}\left({\hat{\theta }}_{k}^{i}|{\hat{\vartheta }}_{j,m}\right)=\frac{1}{{\left(2\pi \right)}^{d/2}|{\hat{\Sigma }}_{j,m}{|}^{1/2}}\exp\left\{-\frac{1}{2}{\left({\hat{\theta }}_{k}^{i}-{\hat{\mu }}_{j,m}\right)}^{T}{\hat{\Sigma }}_{j,m}^{-1}\left({\hat{\theta }}_{k}^{i}-{\hat{\mu }}_{j,m}\right)\right\}, \end{array}$$

where *d* is the dimension of θ, $p\left(z_{i}=j|{\hat{\theta }}_{k}^{i},{\hat{\rho }}_{m}\right)$ denotes the estimated probability that the *j*-th Gaussian component matches the *i*-th PM, ${\hat{\theta }}_{k}^{i}$, under knowing ${\hat{\rho }}_{m}$, and ${\hat{\alpha }}_{j,m}$ and ${\hat{\vartheta }}_{j,m}$ are the *m*-th identification values of weight and mean/covariance corresponding to the *j*-th Gaussian component.

In the M-step, by making the following first derivatives as zero:

(36) $$\begin{array}{cc} \frac{\partial Q(\rho |{\hat{\rho }}_{m})}{\partial \mu_{j}} & =\frac{\partial }{\partial \mu_{j}}\sum_{j=1}^{M}\sum_{i=1}^{N}\left[\ln p_{j}\left({\hat{\theta }}_{k}^{i}|\mu_{j},\Sigma_{j}\right)\right]p\left(j|{\hat{\theta }}_{k}^{i},{\hat{\rho }}_{m}\right)\\ & =\sum_{i=1}^{N}p\left(j|{\hat{\theta }}_{k}^{i},{\hat{\rho }}_{m}\right)\frac{\partial }{\partial \mu_{j}}\ln p_{j}\left({\hat{\theta }}_{k}^{i}|\mu_{j},\Sigma_{j}\right)\\ & =\sum_{i=1}^{N}p\left(j|{\hat{\theta }}_{k}^{i},{\hat{\rho }}_{m}\right){\left({\hat{\theta }}_{k}^{i}-\mu_{j}\right)}^{T}\Sigma_{j}^{-1}=0 \end{array}$$

(37) $$\begin{array}{cc} \frac{\partial Q(\rho |{\hat{\rho }}_{m})}{\partial \Sigma_{j}^{-1}} & =\frac{\partial }{\partial \Sigma_{j}^{-1}}\sum_{j=1}^{M}\sum_{i=1}^{N}\left[\ln p_{j}\left({\hat{\theta }}_{k}^{i}|\mu_{j},\Sigma_{j}\right)\right]p\left(j|{\hat{\theta }}_{k}^{i},{\hat{\rho }}_{m}\right)\\ & =\sum_{i=1}^{N}p\left(j|{\hat{\theta }}_{k}^{i},{\hat{\rho }}_{m}\right)\frac{\partial }{\partial \Sigma_{j}^{-1}}\ln p_{j}\left({\hat{\theta }}_{k}^{i}|\mu_{j},\Sigma_{j}\right)\\ & =\sum_{i=1}^{N}p\left(j|{\hat{\theta }}_{k}^{i},{\hat{\rho }}_{m}\right)\left[\frac{1}{2}\Sigma_{j}-\frac{1}{2}\left({\hat{\theta }}_{k}^{i}-\mu_{j}\right){\left({\hat{\theta }}_{k}^{i}-\mu_{j}\right)}^{T}\right]=0, \end{array}$$

the parameters of ϑ are re-estimated with the updated probabilities:

(38) $${\hat{\mu }}_{j,m+1}=\frac{\sum_{i=1}^{N}p\left(j|{\hat{\theta }}_{k}^{i},{\hat{\rho }}_{m}\right){\hat{\theta }}_{k}^{i}}{\sum_{i=1}^{N}p(j|{\hat{\theta }}_{k}^{i},{\hat{\rho }}_{m})}$$

(39) $${\hat{\Sigma }}_{j,m+1}=\frac{\sum_{i=1}^{N}p\left(j|{\hat{\theta }}_{k}^{i},{\hat{\rho }}_{m}\right)\left({\hat{\theta }}_{k}^{i}-{\hat{\mu }}_{j,m+1}\right){\left({\hat{\theta }}_{k}^{i}-{\hat{\mu }}_{j,m+1}\right)}^{T}}{\sum_{i=1}^{N}p(j|{\hat{\theta }}_{k}^{i},{\hat{\rho }}_{m})}.$$

Identifying α requires optimization of the following term of Equation (32):

$$\sum_{j=1}^{M}\sum_{i=1}^{N}p\left(j|{\hat{\theta }}_{k}^{i},{\hat{\rho }}_{m}\right)\ln\left(\alpha_{j}\right),$$

with the constraint $\sum_{j=1}^{M}\alpha_{j}=1$. However, direct optimization with respect to α is difficult. For simplicity, we equivalently construct:

(40) $$\ell \left(\alpha \right)=\sum_{j=1}^{M}\sum_{i=1}^{N}p\left(j|{\hat{\theta }}_{k}^{i},{\hat{\rho }}_{m}\right)\ln\left(\alpha_{j}\right)+\beta \left(\sum_{j=1}^{M}\alpha_{j}-1\right).$$

Thus, making the first derivative of Equation (40) with respect to α zero, one obtains:

(41) $$\alpha_{j}=\frac{\sum_{i=1}^{N}p\left(j|{\hat{\theta }}_{k}^{i},{\hat{\rho }}_{m}\right)}{-\beta }.$$

Finally, applying the following equation:

$$\sum_{j=1}^{M}\alpha_{j}=\frac{\sum_{j=1}^{M}\sum_{i=1}^{N}p\left(j|{\hat{\theta }}_{k}^{i},{\hat{\rho }}_{m}\right)}{-\beta }=\frac{N}{-\beta }=1,$$

to rearrange Equation (41), gives the weight update:

(42) $${\hat{\alpha }}_{j,m+1}=\frac{1}{N}\sum_{i=1}^{N}p\left(j|{\hat{\theta }}_{k}^{i},{\hat{\rho }}_{m}\right).$$

Summarizing the above, the fit mean, $\mu_{k}^{\theta }$, and covariance, $P_{k}^{\theta }$, of the UI-PM set, ${\hat{\theta }}_{k}$, are obtained as follows:

(43) $$\mu_{k}^{\theta }=\sum_{j=1}^{M}{\hat{\alpha }}_{j,k}{\hat{\mu }}_{j,k}$$

(44) $$P_{k}^{\theta }=\sum_{j=1}^{M}{\hat{\alpha }}_{j}(\Sigma_{j,k}+{\hat{\mu }}_{j,k}{\hat{\mu }}_{j,k}^{T})\;-\;\left(\sum_{j=1}^{M}{\hat{\alpha }}_{j,k}{\hat{\mu }}_{j,k}\right){\left(\sum_{j=1}^{M}{\hat{\alpha }}_{j,k}{\hat{\mu }}_{j,k}\right)}^{T},$$

which corresponds to the output of the EM-PM in Figure 4. Here, ${\hat{\alpha }}_{j,k}$, ${\hat{\mu }}_{j,k}$, and ${\hat{\Sigma }}_{j,k}$ are the terminatively optimized parameters serving for the *j*-th Gaussian component at time *k*.

**Remark** **5.** *EM-PM is initialized by directly computing the stochastic characteristics (average and variance) of ${\hat{\theta }}_{k}$ from multi-sensors in time-domain. Although it may be simple and obvious, such the initialization is in line with the stochastic essence. Moreover, the initialization has no effect on the terminative convergence of the EM-PM, since the convex optimization of Equations (36) and (37) uniquely maximize the expectation.*

## 5. SMCCF

After achieving EM-AM and EM-PM, this section aims to link them and further clarify their input-output relationship with each other. Before doing this, as a comparison, we first show the execution program of directly using the state estimation from the EM-AM output in Figure 7 (as a refinement of the first EM in Figure 4). By using EKF to implement the state estimation computation in the EM-AM, we have:

1. Prediction
  (45) $$\left\{\begin{array}{c} {\hat{x}}_{k|k-1}^{i}=F_{k-1}{\hat{x}}_{k-1}^{i}+M_{k-1}{\hat{a}}_{k}^{i}\\ P_{k|k-1}^{i}=F_{k-1}P_{k-1}^{i}F_{k-1}^{T}+Q_{k-1} \end{array}\right.$$
2. Update
  (46) $$\left\{\begin{array}{c} K_{k}^{i}=P_{k|k-1}^{i}H_{k}^{T}{\left(H_{k}P_{k|k-1}^{i}H_{k}^{T}+R_{k}^{i}\right)}^{-1}\\ {\hat{x}}_{k}^{i}={\hat{x}}_{k|k-1}^{i}+K_{k}^{i}\left(y_{k}^{i}-H_{k}{\hat{x}}_{k|k-1}^{i}-N_{k}{\hat{b}}_{k}^{i}\right)\\ P_{k}^{i}=\left(I-K_{k}^{i}H_{k}\right)P_{k|k-1}^{i} \end{array}\right.,$$
  where ${\hat{\theta }}_{k}^{i}=\left\{{\hat{a}}_{k}^{i},{\hat{b}}_{k}^{i}\right\}$ denotes the identified UI-PM by the EM-AM corresponding to the *i*-th sensor, $F_{k-1}$ and $H_{k}$ are the Jacobian matrices of the dynamic and measurement models. Obviously, in each EM-AM, only the state estimation is corrected by the UI-PM while its covariance computation is the same as that in standard Kalman-based filter. Besides EKF, one has a freedom to select other Kalman-based filters for implementing the state estimation in EM-AM, such as the above-mentioned nonlinear Gaussian filters including UKF, and CKF, among others.

After obtaining *N* groups of $\left\{{\hat{x}}_{k}^{i},{\hat{P}}_{k}^{i}\right\}$, we use the well-known Federal structure to directly fuse $\left\{{\hat{x}}_{k}^{i},{\hat{P}}_{k}^{i}\right\}$ as follows:

(47) $$\left\{\begin{array}{c} {\hat{x}}_{k}^{F}=P_{k}^{F}\left[{\left(P_{k}^{1}\right)}^{-1}{\hat{x}}_{k}^{1}+\cdots +{\left(P_{k}^{N}\right)}^{-1}{\hat{x}}_{k}^{N}\right]\\ P_{k}^{F}={\left[{\left(P_{k}^{1}\right)}^{-1}+\cdots +{\left(P_{k}^{N}\right)}^{-1}\right]}^{-1} \end{array}\right.,$$

where corresponding to Figure 7, the filter weight $\beta_{i}=P_{k}^{F}{\left(P_{k}^{i}\right)}^{-1}$. Of course, there also exist other estimation fusion structures, which have been well studied at present. However, this paper mainly wishes to focus on demonstrating the feasibility and superiority of the simultaneous correction forms in the following (Equation (48)), as compared to the standard mean-based correction form in Equations (45) and (46). Thus, we choose the relatively simple Federal structure for trying to best eliminate the possible influence or interference of fusion on the proposed simultaneous correction here.

The following continues to combine the EM-AM and EM-PM as shown in Figure 8 (which is a refinement of Figure 4). Different from Figure 7, the SMCCF adds the EM-PM between the EM-AM and Federal fusion structures to fit the UI-FTM. Before carrying out fusion, the state estimation and its covariance are simultaneously corrected by the UI-FTM. This means that if the EM-PM fits $\mu_{k}^{\theta }=\left\{\mu_{k}^{a},\mu_{k}^{b}\right\}$ and $P_{k}^{\theta }=\left\{P_{k}^{a},P_{k}^{b}\right\}$ as given in Equations (43) and (44), then employing EKF again as an example, we have:

(48) $$\left\{\begin{array}{c} {\hat{x}}_{k|k-1}^{i}=F_{k-1}{\hat{x}}_{k-1}^{i}+M_{k-1}\mu_{k}^{a}\\ P_{k|k-1}^{i}=F_{k-1}P_{k-1}^{i}F_{k-1}^{T}+M_{k-1}P_{k}^{a}M_{k-1}^{T}+Q_{k-1}^{i}\\ K_{k}^{i}=P_{k|k-1}^{i}H_{k}^{T}{\left(H_{k}P_{k|k-1}^{i}H_{k}^{T}+N_{k}P_{k}^{b}N_{k}^{T}+R_{k}^{i}\right)}^{-1}\\ {\hat{x}}_{k}^{i}={\hat{x}}_{k|k-1}^{i}+K_{k}^{i}\left(y_{k}^{i}-N_{k}\mu_{k}^{b}-H_{k}{\hat{x}}_{k|k-1}^{i}\right)\\ \begin{array}{c} P_{k}^{i}=\left(I-K_{k}^{i}H_{k}\right)P_{k|k-1}^{i}{\left(I-K_{k}^{i}H_{k}\right)}^{T}+\left(K_{k}^{i}N_{k}\right)P_{k}^{b}{\left(K_{k}^{i}N_{k}\right)}^{T}+\;K_{k}^{i}R_{k}{\left(K_{k}^{i}\right)}^{T}. \end{array} \end{array}\right.$$

Again the above-mentioned Federal structure in Equation (47) is used to fuse the state estimation and achieve the SMCCF, denoted as ${\hat{x}}_{k}^{S}$ and ${\hat{P}}_{k}^{S}$. In fact, Figure 7 is based on the standard filter structure in Equations (45) and (46) , where the UI-PM identification values ${\hat{\theta }}_{k}^{i}=\left\{{\hat{a}}_{k}^{i},\;{\hat{b}}_{k}^{i}\right\}$ are directly used for correcting the state prediction and the measurement prediction, regardless of their covariances. But Figure 8 is based on the SMCCF structure in Equation (48), where $\left\{{\hat{\mu }}_{k}^{\theta },\;P_{k}^{\theta }\right\}$ are further mined from ${\hat{\theta }}_{k}^{i}$ by adding the second EM part to simultaneously correct the predictions and the corresponding covariances.

**Remark** **6.** *Of course, there are other execution programs which link the EM-AM and EM-PM to achieve SMCCF. For example, at each iteration of the EM-AM, the output PM identifications by multi-sensors are fit by EM-PM, and fed back to simultaneously correct the state estimation and its covariance. But, this paper mainly aims to demonstrate the superiority of the novel JESI to the standard NES, and SMCCF to the standard nonlinear filters, including EKF, and UKF, among others. The performance analysis or comparison of different fusion structures will be explored in the future work.*

**Remark** **7.** *Maybe, one can find that the state estimation forms between Equations (17), (18), (45), (46), and (48), are slightly different from each other. At each time-recursion, the former is located in the EM-AM by applying these measurements in a sliding window interval [k−l,k] for identifying UI-PM, and further for fitting UI-FTM. Similarly, the latter is located in the fusion center by applying Kalman-based filters for recursively updating the state estimation, which is simultaneously corrected by UI-FTM. Thus, the former needs to be continuously initialized at each recursion as discussed in Remarks 2 and 3, while the latter is only initialized once by ${\hat{x}}_{0}$ at the beginning time.*

### Final Algorithm of SMCCF

Referring to Figure 8 and Figure 9, the final implementation algorithm of SMCCF is as follows.


**Algorithm 1:**
 Step 1: Initialization—${\hat{x}}_{0}$, $P_{0}$ and ${\hat{\theta }}_{0}$ in Equation (27). Step 2: The first-stage EM: Joint state estimation and UI-PM identification. Re-initialization—as discussed in Remarks 2 and 3.Parallel EM-AM of multi-sensors for identifying UI-PMs. Given the measurements $Y_{k-l:k}^{i}$, iteratively compute the analytical UI-PM identification in Equations (22) and (23) until EM-AM terminates ($r>r_{max}$), to finally output a group of UI-PM identification values ${\hat{\theta }}_{k}={\left\{{\hat{\theta }}_{k}^{i}\right\}}_{i=1}^{N}$.Parallel state estimation of multi-sensors. Given the full measurements $Y_{1:k}^{i}$, implement Kalman-based filters for Equation (1) to recursively output *N* groups of $\left\{{\hat{x}}_{k}^{i},P_{k}^{i}\right\}$. As an example the EKF of Equations (45) and (46) (if only corrected by the corresponding UI-PMs ${\hat{\theta }}_{k}={\left\{{\hat{\theta }}_{k}^{i}\right\}}_{i=1}^{N}$). Note that $Y_{k-l}^{k}$ and Equation (26) only service for UI-PM identification. Step 3: UI-FTM fitting.  Re-initialization—average and variance of ${\left\{{\hat{\theta }}_{k}^{i}\right\}}_{i=1}^{N}$ as analyzed in Remark 5.EM-PM: Given a group of ${\left\{{\hat{\theta }}_{k}^{i}\right\}}_{i=1}^{N}$, iteratively compute the fit UI-FTM as shown in Equations (38), (39), and (42) until EM-PM terminates ($m>m_{max}$), to finally output $\mu_{k}^{\theta },\;P_{k}^{\theta }$ of Equation (43).SMCCF: Apply $\left\{\mu_{k}^{\theta },\;P_{k}^{\theta }\right\}$ to simultaneously correct the Kalman-based state estimation $\left\{{\hat{x}}_{k}^{i},\;P_{k}^{i}\right\}$, for example, the EKF of Reference (48). Step 4: Fuse $\left\{{\hat{x}}_{k}^{i},\;P_{k}^{i}\right\}$ by the Federal structure, as given in Equation (47), or other fusion schemes.  Step 5: Repeat Step 2 to Step 4 with time proceeding.

**Remark** **8.** *Compared with the standard NESs, the main factor, affecting the complexity of the SMCCF scheme, Figure 8, lies in the EM-AM iteration times. Owing to the analytical characteristics and to the convex optimization of identifying UI-PM, a direct method of improving EM-AM execution efficiency is to let $r_{max}\equiv 1$; at each time-recursion, EM-AM is executed only once. However, to guarantee accuracy, the measurement may be extended to many samples. On the other hand, compared with the standard EM which only use the UI-mean to correct the orbit state estimation, the SMCCF scheme is slightly more computational complex because it needs EM-PM to further fit the UI-FTM. Besides the above-mentioned differences, the involved fusion structure and orbit state filtering computation in various algorithms are set to be consistent. This allows us to highlight the superiority of the SMCCF design as compared with the standard NES and EM algorithms, and further facilitates the following demonstrations of the different algorithms under a uniform benchmark by eliminating the interference of other factors.*

## 6. Simulation

This section considers the space target orbit estimation with the $J_{2}$ perturbation. Let the target’s position and velocity characterize the system’s state, i.e., $x={\left[\begin{array}{cccccc} \lambda & \varphi & h & v_{\lambda } & v_{\varphi } & v_{h} \end{array}\right]}^{T}$, then the dynamic equation is:

(49) $$\left\{\begin{array}{c} \dot{\lambda }=v_{\lambda }\\ \dot{\varphi }=v_{\varphi }\\ \dot{h}=v_{h}\\ {\dot{v}}_{\lambda }=-\frac{\mu }{r^{3}}\lambda +\frac{\mu }{r^{3}}J_{2}{\left(\frac{R_{e}}{r}\right)}^{2}\lambda \left(7.5\frac{h^{2}}{r^{2}}-1.5\right)\\ {\dot{v}}_{\varphi }=-\frac{\mu }{r^{3}}\varphi +\frac{\mu }{r^{3}}J_{2}{\left(\frac{R_{e}}{r}\right)}^{2}\varphi \left(7.5\frac{h^{2}}{r^{2}}-1.5\right)\\ {\dot{v}}_{h}=-\frac{\mu }{r^{3}}h+\frac{\mu }{r^{3}}J_{2}{\left(\frac{R_{e}}{r}\right)}^{2}h\left(7.5\frac{h^{2}}{r^{2}}-4.5\right) \end{array}\right.$$

where the target velocity is directly disturbed by the $J_{2}$ perturbation, which further and indirectly affects the target position. If we view the orbit perturbation as a UI coupled with the target state, then the above dynamic equations can be further simplified and rearranged as:

(50) $$\dot{x}\left(t\right)=F\left(t\right)x\left(t\right)+M\theta \left(t\right)+w\left(t\right)$$

where $F\left(t\right)=\left[\begin{array}{cccccc} 0 & 0 & 0 & 1 & 0 & 0\\ 0 & 0 & 0 & 0 & 1 & 0\\ 0 & 0 & 0 & 0 & 0 & 1\\ -\frac{\mu }{r^{3}} & 0 & 0 & 0 & 0 & 0\\ 0 & -\frac{\mu }{r^{3}} & 0 & 0 & 0 & 0\\ 0 & 0 & -\frac{\mu }{r^{3}} & 0 & 0 & 0 \end{array}\right]$ $M=\left[\begin{array}{ccc} 0 & 0 & 0\\ 0 & 0 & 0\\ 0 & 0 & 0\\ 1 & 0 & 0\\ 0 & 1 & 0\\ 0 & 0 & 1 \end{array}\right]$ $\theta \left(t\right)={\left[\begin{array}{ccc} a\left(x\right) & b\left(x\right) & c\left(x\right) \end{array}\right]}^{T}$

with

$$\left\{\begin{array}{c} a\left(x\right)=\frac{\mu }{r^{3}}J_{2}{\left(\frac{R_{e}}{r}\right)}^{2}\lambda \left(7.5\frac{h^{2}}{r^{2}}-1.5\right)\\ b\left(x\right)=\frac{\mu }{r^{3}}J_{2}{\left(\frac{R_{e}}{r}\right)}^{2}\varphi \left(7.5\frac{h^{2}}{r^{2}}-1.5\right)\\ c\left(x\right)=\frac{\mu }{r^{3}}J_{2}{\left(\frac{R_{e}}{r}\right)}^{2}h\left(7.5\frac{h^{2}}{r^{2}}-4.5\right) \end{array}\right..$$

In Equation (50), F(t) is the transfer matrix of the orbit state, θ denotes the orbit perturbation which can be understood as an unknown input coupled with the state, and *M* is the multiplicative matrix serving for θ. Indeed, Equation (50) is equivalently derived from Equation (49) and the derivation process is relatively simple so we omit it and only give the result. The reason of deriving Equation (50) from Equation (49) lies in that Equation (50) has the same functional structure as Equation (1), which facilitates application of the SMCCF algorithm for dealing with the practical orbit state estimation issue.

Discretizing Equation (50) by the fourth-order Runge-Kutta yields:

(51) $$x_{k+1}=\left(I+TF_{k}+\frac{T^{2}}{2}F_{k}^{2}+\frac{T^{3}}{6}F_{k}^{3}+\frac{T^{4}}{24}F_{k}^{4}\right)x_{k}+M\theta_{k}+w_{k},$$

where *T* is the sampling interval. The measurement equation to observe the space target is:

(52) $$y_{k}=\sqrt{{\left(\lambda_{k}-\lambda_{0}\right)}^{2}+{\left(\varphi_{k}-\varphi_{0}\right)}^{2}+{\left(h_{k}-h_{0}\right)}^{2}}+v_{k}=h\left(x_{k}\right)+v_{k},$$

where ($\lambda_{0}$, $\varphi_{0}$, $h_{0}$) denote the sensor location. By setting different ($\lambda_{0}$, $\varphi_{0}$, $h_{0}$), multi-sensors observation system can be established.

The model parameters are set as follows:

- *T* = 1 s
- Earth’s mass, $M=5.965\times 10^{24}$ (kg)
- Gravitational constant, *G* = 6.67259 ×$10^{-11}$(N · m${}^{2}$/kg${}^{2}$)
- Earth’s gravitational constant, μ=GM
- Earth’s radius, Re = 6,874,140 (m), $J_{2}$ = $1.0826269\times 10^{-3}$
- The simulation sampling length, *K* = 5000 s
- The true and initial orbit state,
  $\begin{array}{c} x_{0}=\begin{array}{cccccc} {}[4.590\times 10^{6} & 4.388\times 10^{6} & 3.228\times 10^{6} & -4.612\times 10^{3} & 5.014\times 10^{2} & 5.876\times 10^{3} \end{array}{]}^{T} \end{array}$
- The covariances of $w_{k}$ and $v_{k}$,
  $\begin{array}{c} Q_{k}=diag\left(\begin{array}{cccccc} {\left(10^{-2}\right)}^{2} & {\left(10^{-2}\right)}^{2} & {\left(10^{-2}\right)}^{2} & {\left(6\times 10^{-3}\right)}^{2} & {\left(6\times 10^{-3}\right)}^{2} & {\left(6\times 10^{-3}\right)}^{2} \end{array}\right),\;R_{k}=10^{2} \end{array}$

The simulated tracking parameters are set as follows: the window length l=2, five iterations are employed for EM-AM, and 30 for EM-PM, and the number of sensors and GM models are 10 and 5, respectively. Finally, our simulation results are obtained by running ten independent Monte Carlo runs. The initial orbit state estimation is:

${\hat{x}}_{0}=x_{0}+{\left[\begin{array}{cccccc} 400\; & \;400\; & \;400\; & \;0.8\; & \;0.8\; & \;0.8 \end{array}\right]}^{T}$

$P_{0}=diag\left(\begin{array}{cccccc} 400^{2}\; & \;400^{2}\; & \;400^{2}\; & \;0.8^{2}\; & \;0.8^{2}\; & \;0.8^{2} \end{array}\right)$.

For spatial scales, define the root mean square errors (RMSE)s of position and velocity at time *k* as:

(53) $$\begin{array}{c} RMSE_{pos}\left(k\right)=\sqrt{\frac{1}{N}\sum_{mc=1}^{N}\left({\left(\lambda_{k}^{mc}-{\hat{\lambda }}_{k}^{mc}\right)}^{2}+{\left(\varphi_{k}^{mc}-{\hat{\varphi }}_{k}^{mc}\right)}^{2}+{\left(h_{k}^{mc}-{\hat{h}}_{k}^{mc}\right)}^{2}\right)} \end{array}$$

(54) $$\begin{array}{c} RMSE_{vel}\left(k\right)=\sqrt{\frac{1}{N}\sum_{mc=1}^{N}\left({\left(v_{\lambda ,k}^{mc}-{\hat{v}}_{\lambda ,k}^{mc}\right)}^{2}+{\left(v_{\varphi ,k}^{mc}-{\hat{v}}_{\varphi ,k}^{mc}\right)}^{2}+{\left(v_{h,k}^{mc}-{\hat{v}}_{h,k}^{mc}\right)}^{2}\right)}. \end{array}$$

For the time scale, define the RMSEs as:

(55) $${\bar{RMSE}}_{pos}=\sqrt{\frac{1}{K}\sum_{k=1}^{K}RMSE_{pos}\left(k\right)}$$

(56) $${\bar{RMSE}}_{vel}=\sqrt{\frac{1}{K}\sum_{k=1}^{K}RMSE_{vel}\left(k\right)},$$

where *N* is the independent Monte Carlo running times and *K* is the simulation sampling length.

**Figure 10:**
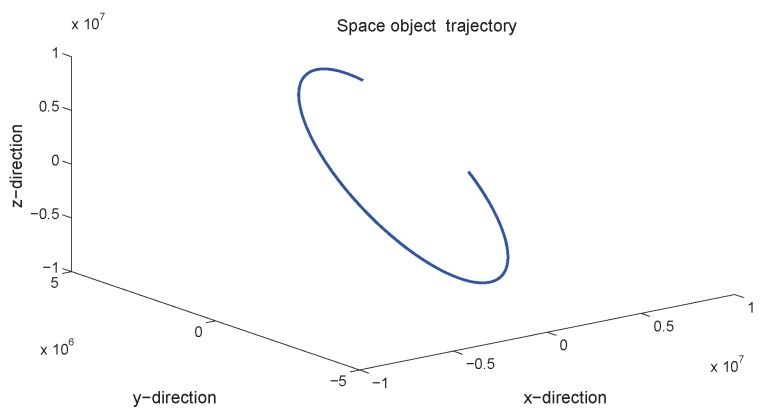
Space target trajectory.

**Figure 11:**
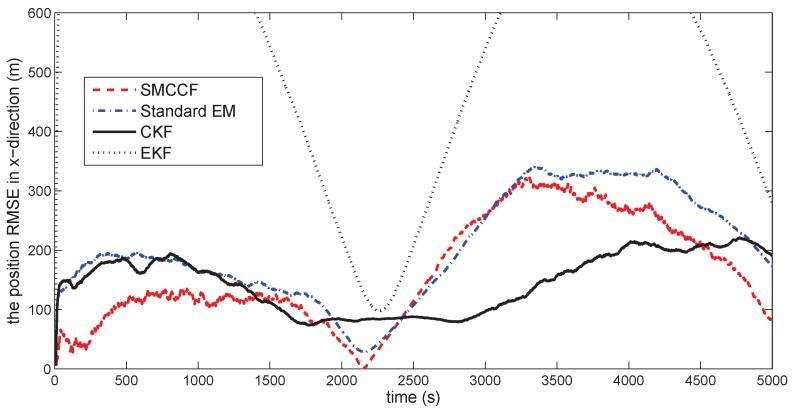
The position RMSE in the *x*-direction.

**Figure 12:**
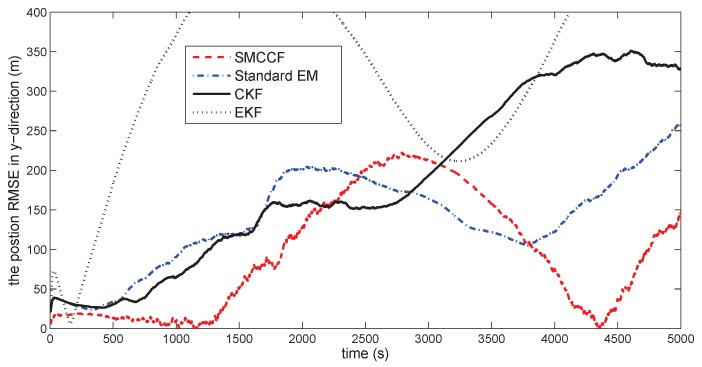
The position RMSE in the *y*-direction.

**Figure 13:**
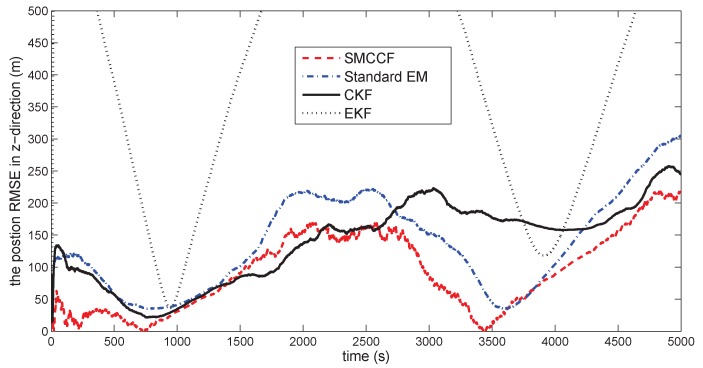
The position RMSE in the *z*-direction.

**Figure 14:**
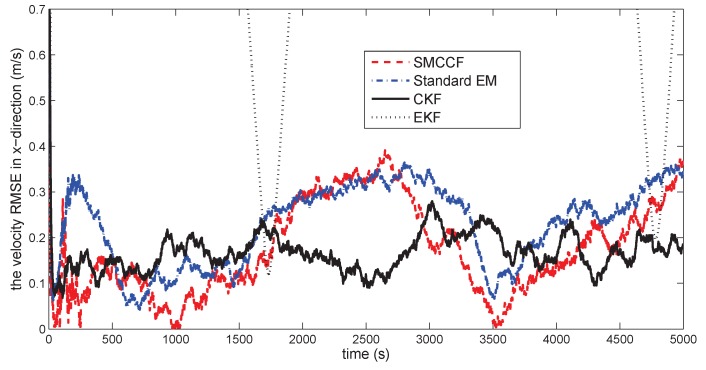
The velocity RMSE in the *x*-direction.

**Figure 15:**
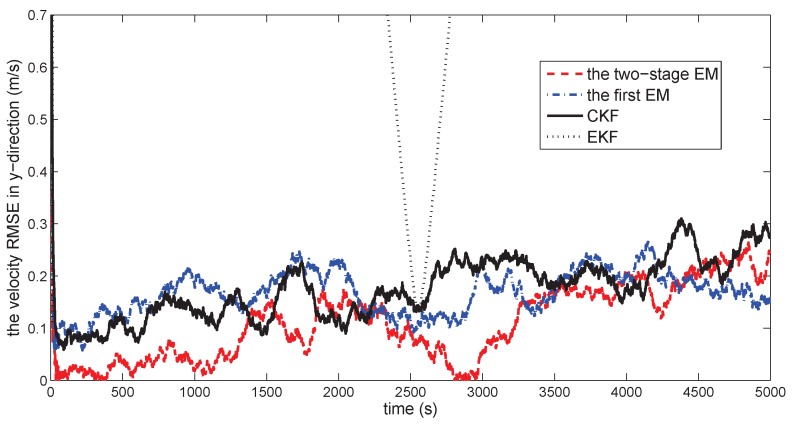
The velocity RMSE in the *y*-direction.

**Figure 16:**
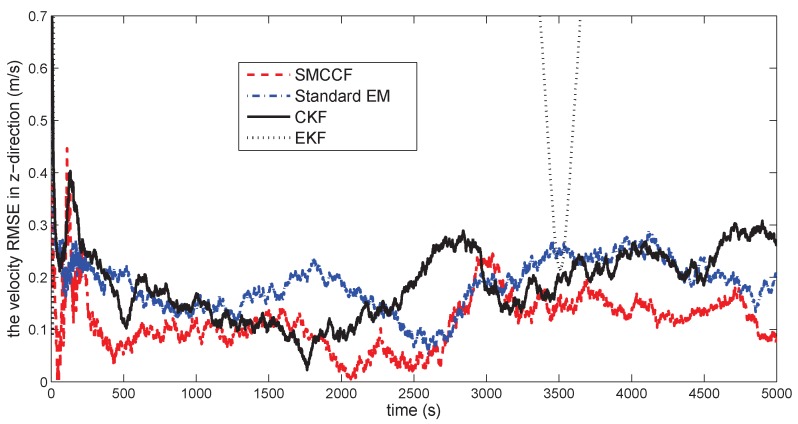
The velocity RMSE in the *z*-direction.

Figure 10 gives the space target trajectory. In Figure 11, Figure 12, Figure 13, Figure 14, Figure 15 and Figure 16, all the involved algorithms are obtained by fusing the state estimation results from multiple sensors under the Federal fusion structure. The proposed SMCCF obviously outperforms the other methods, which demonstrates the feasibility and effectiveness of the two-stage EM design scheme in SMCCF, as compared to the standard EM and NES schemes. The $J_{2}$ perturbation directly affects and acts on the orbit velocity. Wonderfully, the velocity estimation performance is well improved throughout the entire sampling interval, which just demonstrates the superiority of the novel SMCCF in coping with the perturbation. The above results can also be verified by Table 1, which computes the $RMSE_{pos}$ and $RMSE_{vel}$. The velocity estimation accuracy performance is improved by 23.4%, as compared to the standard EM, and the position performance is improved by 19.4%.

## 7. Conclusions

In the case that a UI is associated with the system state, UI estimation requires the FTM at least. For these dynamic systems with the above-considered UI forms, this paper proposes a novel SMCCF based on a two-stage EM framework, which further improves the state estimation performance by fitting the UI-FTM and using it to simultaneously correct the state estimation and its covariance. Through a space target orbit tracking example, the superiority of SMCCF, as compared to the standard NESs or EM algorithms, is demonstrated. 

## Figures and Tables

**Table 1:** RMSE comparison.

Algorithms	EKF	CKF	Standard EM	SMCCF
RMSE${}_{pos}$	856.0445	329.0306	310.5345	250.2542
RMSE${}_{vel}$	4.7096	0.3743	0.3584	0.2745
